# STAT3-Specific Single Domain Nanobody Inhibits Expansion of Pathogenic Th17 Responses and Suppresses Uveitis in Mice

**DOI:** 10.3389/fimmu.2021.724609

**Published:** 2021-09-15

**Authors:** Evaristus C. Mbanefo, Ming Yan, Minkyung Kang, Sahar A. Alhakeem, Yingyos Jittayasothorn, Cheng-Rong Yu, Ashutosh Parihar, Sunanda Singh, Charles E. Egwuagu

**Affiliations:** ^1^Molecular Immunology Section, Laboratory of Immunology, National Eye Institute (NEI), National Institutes of Health (NIH), Bethesda, MD, United States; ^2^Immunoregulation Section, Laboratory of Immunology, NEI, NIH, Bethesda, MD, United States; ^3^Singh Biotechnology, Tampa Bay, FL, United States

**Keywords:** nanobody, STAT3, Th17, uveitis, EAU, Th1

## Abstract

STAT3 activates transcription of genes that regulate cell growth, differentiation, and survival of mammalian cells. Genetic deletion of *Stat3* in T cells has been shown to abrogate Th17 differentiation, suggesting that STAT3 is a potential therapeutic target for Th17-mediated diseases. However, a major impediment to therapeutic targeting of intracellular proteins such as STAT3 is the lack of efficient methods for delivering STAT3 inhibitors into cells. In this study, we developed a novel antibody (SBT-100) comprised of the variable (V) region of a STAT3-specific heavy chain molecule and demonstrate that this 15 kDa STAT3-specific nanobody enters human and mouse cells, and induced suppression of STAT3 activation and lymphocyte proliferation in a concentration-dependent manner. To investigate whether SBT-100 would be effective in suppressing inflammation *in vivo*, we induced experimental autoimmune uveitis (EAU) in C57BL/6J mice by active immunization with peptide from the ocular autoantigen, interphotoreceptor retinoid binding protein (IRBP_651-670_). Analysis of the retina by fundoscopy, histological examination, or optical coherence tomography showed that treatment of the mice with SBT-100 suppressed uveitis by inhibiting expansion of pathogenic Th17 cells that mediate EAU. Electroretinographic (ERG) recordings of dark and light adapted a- and b-waves showed that SBT-100 treatment rescued mice from developing significant visual impairment observed in untreated EAU mice. Adoptive transfer of activated IRBP-specific T cells from untreated EAU mice induced EAU, while EAU was significantly attenuated in mice that received IRBP-specific T cells from SBT-100 treated mice. Taken together, these results demonstrate efficacy of SBT-100 in mice and suggests its therapeutic potential for human autoimmune diseases.

## Introduction

Cytokines such as IFN-γ, IL-2, IL-4, IL-6, IL-10, IL-21, IL-23, IL-27, and IL-35 that regulate immune responses and autoimmune diseases mediate their biological activities through the activation of the Janus Kinase (JAK)/STAT pathway (1, 2). This evolutionary conserved signal transduction pathway is orchestrated by the 4 Janus kinases (Jak1, Jak2, Jak3, Tyk2) and the 7-member signal transducer and activator of transcription factor (STAT) family of proteins, STAT1, STAT2, STAT3, STAT4, STAT5a, STAT5b, and STAT6. Binding of a cytokine to its cognate receptor activates the requisite Jak proteins by transphosphorylation, providing docking sites for recruitment of specific STATs. STATs recruited to the receptor complex are phosphorylated at a critical tyrosine residue, form homo- or hetero-dimers, and translocate into the nucleus where they bind to specific DNA sequences and activate gene transcription. Thus, the JAK/STAT pathway provides a rapid membrane to nucleus mechanism that transduces signals from the cell membrane to the nucleus and couples specific gene expression to change in the behavior of the cell (2).

STAT3 is unique among STAT proteins because it plays an essential and non-redundant role in mammalian cells. In mice, genetic deletion of *Stat3* results in embryonic lethality and mice die within 3 weeks after birth. Dominant-negative mutations in the DNA-binding domain of STAT3 are the cause of the rare immunodeficiency disorder known as Job’s syndrome for which there is no cure and this hyper-IgE syndrome is characterized by recurring skin and lung infections, high risk of breaking bones, and development of muscular skeletal diseases (3, 4). Although much is known about the role of aberrant activation of STAT3 which results in uncontrolled proliferation, cell growth, and oncogenesis, STAT3 has wide-ranging functions in T-cells and serves as a convergence point for mechanisms that regulate lymphocyte quiescence and those controlling T-cell activation and survival. In contrast to its role in promoting proliferation of activated T cells, it maintains T cells at the G0 phase of the cell cycle by binding *FoxO1* or *FoxO3a* promoter and up-regulating the expression of these Class-O Forkhead transcription factors, which play essential roles in maintaining T-cells in the quiescent state (5). Furthermore, STAT3-deficiency in T cells results in downregulation of *FoxO1, FoxO3a* and marked decrease of *FoxO*-target genes such as *IkB* and *p27Kip1* leading to enhancement of NF-kB activation and production of IL-2 (5, 6). On the other hand, STAT3 is required for activation of Th17 master transcription factor, RORγt, and the expression of its signature proinflammatory cytokine IL-17. Importantly, mice with targeted deletion of STAT3 in CD4^+^ T cells are resistant to development of experimental autoimmune uveitis (EAU) and experimental autoimmune encephalomyelitis (EAE) (7, 8), indicating that STAT3 is a potential therapeutic target for these central nervous system (CNS) autoimmune diseases and other autoinflammatory diseases.

STAT3 is a relatively undruggable therapeutic target and not easily targeted pharmacologically because it is an intracellular protein. Several noninvasive methods have been used to deliver STAT3 mimetic peptides coupled to membrane permeable hydrophobic lipophilic motifs to specifically inhibit STAT3 SH2 domains or binding of STAT3 to kinase inhibitory sites on Jak or cytokine receptors with varying degrees of success. In this study, we have used a miniature Ab developed by Singh Biotechnology which consists of a single VHH derived from a camelid immunoglobulin heavy chain variable region devoid of light chain and demonstrated capacity of this nanobody, SBT-100, to penetrate lymphocytes and inhibit IL-6/STAT3 signaling pathways of primary mouse CD4^+^ lymphocytes and human Jurkat T cells. We provide evidence that SBT-100 is also effective *in vivo* and suppresses the development of EAU by inhibiting the expansion of pathogenic Th17 cells.

## Materials and Methods

### Mice and Reagents

Six- to eight-week old C57BL/6J mice were purchased from Jackson Laboratory (Jackson Laboratory, Bar Harbor, ME). Animals were housed at the NIH/NEI animal facility, and maintained under light-dark cycle with unlimited access to water and chow. All animal care and procedures were humane and conformed with the National Institute of Health guidelines (NIH Animal Care and Use Committee guidelines). The experiments were approved under the NIH/NEI Animal Study Protocol (ASP# NEI-597). SBT-100 was developed by Singh Biotechnology (Tampa Bay, FL).

### Cells and Cell Culture

Jurkat cells, Clone E6-1 (ATCC^®^ TIB-152^™^) were obtained from ATCC (Gaithersburg, MD). All cells were cultured in complete RPMI 1640 media (supplemented with fetal bovine serum [FBS]) to a final concentration of 10% and 1% penicillin-streptomycin, 2mM L-glutamine (Life Technologies, Grand Island, NY), 5µM 2-mercaptoethanol in a humidified incubator at 37°C and 5% CO_2_. IL-6 was used at a concentration of 10 ng/mL (R&D Systems, Minneapolis, MN).

### Experimental Autoimmune Uveitis

EAU was induced in C57BL/6J mice by active immunization with IRBP_651-670_-peptide (300 µg per mouse) in a 200µL emulsion (1:1 v/v) with complete Freund’s adjuvant (CFA) containing *Mycobacterium tuberculosis* strain H37RA (2.5 mg/mL) subcutaneously as previously described (9). Mice also received intraperitoneal injection of *Bordetella pertussis* toxin (1 µg/mouse) concurrently with immunization. Starting from Day -1 of immunization to Day 12 postimmunization, mice were treated twice daily by i.p. injection with either 100 µL PBS or SBT-100 (10 mg/kg body weight in 100 µL PBS). For each study, the mice were age and sex matched and 4-8 mice were used per group. Clinical disease was established and scored by fundoscopy and histology as described previously (9). Eyes were examined for disease severity using binocular microscope with coaxial illumination. Eyes for histology were enucleated 21 days postimmunization, fixed in 10% buffered formalin, and serially sectioned in the vertical pupillary-optic nerve plane. All sections were stained with hematoxylin and eosin (10).

### Adoptive Transfer

EAU was induced in wild-type C57BL/6J mice by active immunization with IRBP_651-670_ in CFA as described above Experimental Autoimmune Uveitis section. Starting from Day -1 of immunization to Day 12 postimmunization, mice were treated twice daily by i.p. injection with either 100 µL PBS or SBT-100 (10 mg/kg body weight in 100 µL PBS). Mice exhibiting clinical features of uveitis by day 21 postimmunization were identified by funduscopic examination. Cells from the lymph nodes and spleen of control or SBT-100-treated mice with EAU were reactivated for 72 h at 2×10^6^ cells/ml in medium containing 20 µg/ml IRBP_651-670_. The activated live cells were purified by centrifugation over Lympholyte M (Cedarlane, Burlington, NC), washed with PBS and 3×10^7^ cells were transferred i.p. to naïve WT C57BL/6J mice. Twelve days after cell transfer, disease was assessed by fundoscopy, histopathology, OCT, and ERG as described (9).

### Fundoscopy

Fundoscopic examinations were performed at day 10 to 21 after EAU induction. Following administration of systemic anesthesia [intraperitoneal injection of ketamine (1.4 mg/mouse) and xylazine (0.12 mg/mouse)], the pupil was dilated by topical administration of 1% tropicamide ophthalmic solution (Alcon Inc., Fort Worth, TX). Fundus image was captured using Micron III retinal imaging microscope (Phoenix Research Labs) for small rodent or a modified Karl Storz veterinary otoendoscope coupled with a Nikon D90 digital camera, as previously described (11). At least 6 images (2 posterior central retinal view, 4 peripheral retinal views) were taken from each eye by positioning the endoscope and viewing from superior, inferior, lateral, and medial fields and each individual lesion was identified, mapped, and recorded. The clinical grading system for retinal inflammation was as established (9, 12).

### Histological Analysis

For histology, eyes were enucleated on Day 21 postimmunization, fixed in 10% buffered formalin, specimens were dehydrated through graded alcohol and embedded in paraffin. Serial vertical sections through the papillary-optic nerve plane were cut and stained with hematoxylin and eosin (H&E). Clinical scores and assessments of disease severity were based on pathological changes at the optic nerve disc and retinal tissues as previously described (11, 13). Photographs of representative sections were taken on a Zeiss photomicroscope.

### Spectral-Domain Optical Coherence Tomography

Optical coherence tomography (OCT) is a noninvasive procedure that allows visualization of internal microstructure of various eye structures in living animals and was performed as previously described (9). An SD-OCT system with 820 nm center wavelength broadband light source (Bioptigen, NC) was used for *in vivo* non-contact imaging of eyes from control or EAU mice. Mice were anesthetized and the pupils dilated. Mice were then immobilized using an adjustable holder that could be rotated easily allowing for horizontal or vertical scanning and each scan was performed at least twice, with realignment each time. The dimension of the scan (in depth and transverse extent) was adjusted until the optimal signal intensity and contrast were achieved. Retinal thickness was measured from the central retinal area of all images obtained from both horizontal and vertical scans from the same eye, using the system software, and averaged. The method used to determine the retinal thicknesses in the system software was as described (14).

### Electroretinogram

Before the ERG recordings, mice were dark-adapted overnight, and experiments were performed under dim red illumination as previously described (9). Mice were anesthetized with a single intraperitoneal injection of ketamine (1.4 mg/mouse) and xylazine (0.12 mg/mouse) and pupils were dilated with Mydriacyl containing 0.5% tropicamide and 0.5% phenylephrine hydrochloride (Santen Pharmaceutical Co., Osaka, Japan). ERGs were recorded using an electroretinography console (Espion E2; Diagnosys LLC, Lowell, MA) that generated and controlled the light stimulus. Dark-adapted ERG was recorded with a single flash delivered in a Ganzfeld dome with intensity of -4 to 1 log cd·s/m^2^ delivered in 7 steps. Light-adapted ERG was obtained with a 10 cd·s/m^2^ background, and light stimuli started at 0.3 to 100 cd·s/m^2^ in 6 steps. Gonioscopic prism solution (Alcon Labs, Fort Worth, TX) was used to provide good electrical contact and to maintain corneal moisture. A reference electrode (gold wire) was placed in the mouth, and a ground electrode (subcutaneous stainless-steel needle) was positioned at the base of the tail. Signals were differentially amplified and digitized at a rate of 1 kHz. Amplitudes of the major ERG components (a- and b-wave) were measured (Espion software; Diagnosys LLC) using automated and manual methods. Immediately after ERG recording, imaging of the fundus was performed as previously described (11).

### Retinal Cells Isolation

To characterize infiltrating inflammatory cells in the retina of EAU mice, mice were euthanized and perfused with PBS as described (9). Enucleated eyes were put in a petri dish containing culture medium and the retina isolated under a dissecting microscope by cutting along the limbus and lens, and the cornea was carefully removed. The retina was then peeled off and transferred to RPMI media containing collagenase (1mg/mL) and DNase (10µg/mL) for 2 h at 37°C. The digesting tissue was periodically pipetted every 30 min to enhance tissue digestion. The digestion was stopped by adding tenfold volume of complete medium. The cells were then washed twice with complete media and cells counted using the Vi-Cell XR cell viability analyzer (Beckman Coulter).

### Preparation of Single Cell Suspension of Draining Lymph Nodes and Spleen

DLNs and spleens were dissected, and cells freed by teasing in a 40µm pore cell strainer. Following washing in RPMI 1640 medium, erythrocytes were lysed using 5 mL of ACK RBC lysis buffer (Quality Biological, MD) for 3 min. The lysis was stopped by adding 10× volume of the medium. Following two washes, cells were resuspended and seeded at a concentration of 2 × 10^6^/mL.

### Intracellular Cytokine Staining and FACS Analysis

For intracellular cytokine detection, cells were restimulated for 5 h with PMA (50 ng/ml)/ionomycin (500 ng/ml). GolgiPlug was added in the last 3 h and intracellular cytokine staining was performed using BD Biosciences Cytofix/Cytoperm kit as recommended (BD Pharmingen, San Diego, CA). FACS analysis was performed on a CytoFLEX Flow Cytometry Instrument (Beckman Coulter, Indianapolis, IN) using protein-specific monoclonal antibodies and corresponding isotype control Abs (BD Pharmingen, San Diego, CA) as previously described (9). FACS analysis was performed on samples stained with mAbs conjugated with fluorescent dyes and each experiment was color compensated. Dead cells were stained with dead cell exclusion dye (Fixable Viability Dye eFluor^®^ 450; eBioscience). Gates were set using isotype controls.

### Detection of Cytokine Secretion by ELISA

CD4^+^ T cells from the LN of EAU mice that were treated with PBS or SBT-100 were restimulated *in vitro* with 20 µg/mL IRBP_651-670_-peptide. Supernatants were collected and cytokine levels quantified using Quantikine ELISA as recommended by the manufacturer.

### Cytokine Quantification by Multiplex ELISA

Plasma from mice was separated by centrifugation at 1000 × g for 10 min and the cytokines were quantified using LEGENDplex Mouse Inflammation Panel as recommended by the manufacturer (BioLegend, San Diego, CA). Data acquisition was performed on CytoFLEX Flow Cytometer (Beckman Coulter, Indianapolis, IN) and analyzed using BioLegend’s LEGENDplex™ data analysis software.

### Lymphocyte Proliferation Assay

Primary CD4^+^ T cells or human Jurkat cells were activated with anti-CD3/anti-CD28 antibodies for 48 h or 72 h with SBT-100 at 50-100 µg/mL or PBS. The cells were then washed and pulsed with ^3^H-thymidine (0.5 µCi/10 µl/well) for 48-72 h and analyzed as described (5). Presented data are mean CPM ± S.E. of responses of six replicate cultures.

### Western Blot Detection of pSTAT3

Cell extracts (20-40 µg/lane) were fractionated on 4-12% gradient SDS-PAGE in reduced condition and Western blot analysis was performed using antibodies specific to mouse STAT3, pSTAT3, or β-actin (Cell Signaling Technology, Danvers, MA). The primary antibodies were detected using anti-mouse-IRDye 680RD and anti-rabbit-IRDye 800RD secondary antibodies for the Li-Cor two-color system, which allows detection of two targets on the same membrane. Each Western blotting analysis was repeated at least three times. pSTAT3 levels were normalized to total β-actin and quantified using Image-J software.

### Statistics

Data analysis and graph plots were performed using GraphPad Prism 8, using two-tailed unpaired Student’s *t* test for pairwise comparisons or one-way ANOVA with multiple pairwise *t* test, depending on the experiments. Data are shown as mean and SD and statistical significance for inferences was based on *p* < 0.05. Asterisks in figures denote *p*-values (*p < 0.05, **p < 0.01, ***p < 0.001, ****p < 0.0001).

## Results

### SBT-100 Suppresses T Cell Proliferation and STAT3 Activation in Primary T Cells

**Figure 1:**
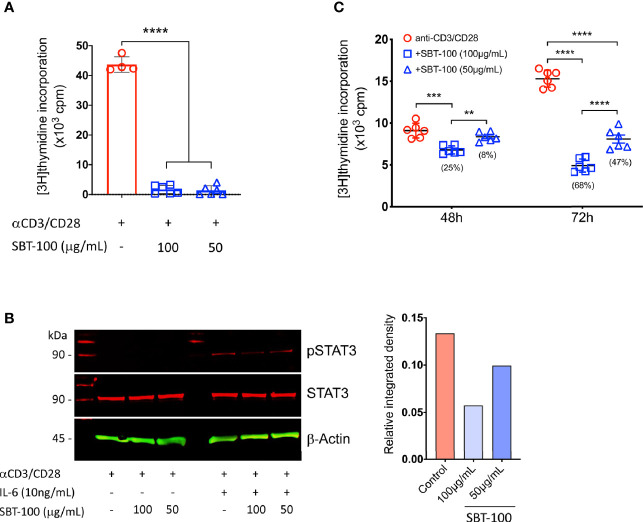
SBT-100 inhibits lymphocyte and STAT3 activation. **(A)** Sorted mouse primary naïve CD4^+^ T cells were stimulated with anti-CD3/CD28 in medium containing SBT-100 and on Day 3 T cell proliferation was assessed by [3H]-thymidine incorporation assay. **(B)** Cells stimulated for 48 h were removed, washed, and starved for 2 h in serum-free medium containing 0.5% BSA and then stimulated for 30 min with IL-6 at 10 ng/ml. Whole cell lysates from the cells were analyzed by Western blotting and pSTAT3 levels were normalized to total β-actin and quantified using Image-J software. **(C)** Human Jurkat T cells were stimulated for 48 h or 72 h with anti-CD3/CD28 in medium containing SBT-100 and on Day 3 T cell proliferation was assessed by [3H]-thymidine incorporation assay. Data represent at least three independent experiments and presented as mean ± SEM. (**p < 0.01; ***p < 0.001; ****p < 0.0001).

Previous studies have shown that activation of STAT3 pathway regulates T cell proliferation and Th17 differentiation while loss of STAT3 in T cells prevents development of CNS autoimmune diseases (7, 8, 15). In this study, we used a proprietary antibody developed to penetrate membranes, the blood-brain barrier (BBB), and the blood retina barrier (BRB) to examine whether this camelid-derived nanobody, SBT-100, would be effective in suppressing Th17-induced autoimmune diseases. SBT-100 is a single domain 2.5 nm, 15 kDa antibody comprised of a single monomeric variable antibody domain (sdAb) and lacks the light chain and CH domain of the heavy chain normally present in the conventional Fab region (16). To determine whether SBT-100 can enter the cytoplasm of primary T cells and antagonize STAT3 signaling, we isolated cells from the spleen of C57BL/6J mice, sorted T cells using CD4-specific magnetic beads, and the cells were shown to be CD4^+^ lymphocytes by FACS analysis. To determine whether SBT-100 can inhibit STAT3 activation, we stimulated the cells with anti-CD3/CD28 for 3 days in medium containing SBT-100 (100 µg/ml or 50 µg/ml). Analysis of the cells by [^3^H]-thymidine incorporation and lymphocyte proliferation assay revealed significant inhibition of T cell proliferation (**Figure 1A**). For evaluating STAT3 activation, after 48 h of stimulation with anti-CD3/anti-CD28, the cells were washed, starved for 2 h in serum-free medium containing 0.5% BSA, and restimulated for 30 min with IL-6 (10ng/ml). Western blot analysis of the lysates revealed that in the absence of SBT-100, the IL-6 activated STAT3 (pSTAT3) while the addition of SBT-100 suppressed pSTAT3 activation in a concentration-dependent manner (**Figure 1B**). We also demonstrated that the inhibitory effect of SBT-100 suppressed proliferation of human Jurkat T cells as indicated by [^3^H]-thymidine incorporation and lymphocyte proliferation assay (**Figure 1C**).

### SBT-100 Ameliorates Uveitis and Preserves Vision During Intraocular Inflammation

**Figure 2:**
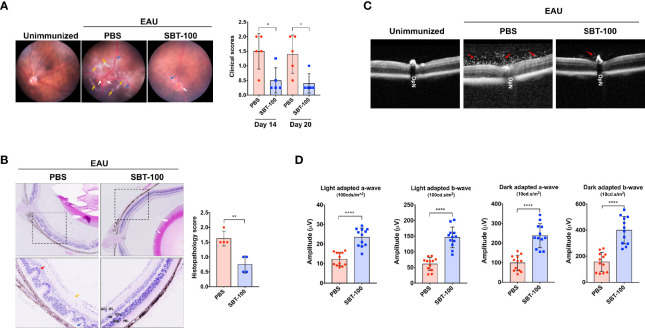
SBT-100 ameliorates experimental autoimmune uveitis (EAU). C57BL/6J mice were immunized with IRBP_651-670_ in CFA and treated with PBS or SBT-100 and development of EAU was assessed by fundoscopy **(A)**, histology **(B)**, OCT **(C)**, or ERG **(D)**. Fundus images reveal inflammation with blurred optic disc margins and enlarged juxtapupillary area (white arrows), retinal vasculitis (blue arrows), and yellow-whitish retinal and choroidal infiltrates (orange arrows). Clinical scores and assessment of disease severity were based on changes at the optic nerve disc or retinal vessels and retinal and choroidal infiltrates (n = 5; from 5 mice). There were characteristic extensive retinal lesions with some confluent lesions due to inflammatory cell infiltration (yellow arrow), blurry optic disc margin (white arrow), and vasculitis (blue arrow) in the untreated EAU control mice, all of which were mild or absent in the SBT-100 treated mice. EAU score was significantly higher in the PBS-treated group as compared to the SBT-100 treated group. Histopathology of a cross-section of the eye was performed on Day 21 postimmunization (n=4; from 4 mice). Clinical and histopathology score guidelines were as previously reported. **(C)** OCT images show accumulation of infiltrating cells around the optic nerve and damage to the optic disc (red arrow). **(D)** Light-adapted (100 cd.s/m^2^) and dark-adapted (10 cd.s/m^2^) a- and b-waves on Day 18 postimmunization were significantly lower in the untreated mice compared to the SBT-100 treated group (n=12; 2 eyes from 6 mice). OpN, optic nerve; GCL, ganglion cell layer; INL, inner nuclear layer; ONL, outer nuclear layer; IPL, inner plexiform layer; OPL, outer plexiform layer; PRL, photoreceptor layer. Data represent at least three independent experiments and presented as mean ± SEM. (*p < 0.05; **p < 0.01; ****p < 0.0001).

Experimental autoimmune uveitis (EAU) is a predominantly T-cell-mediated CNS autoimmune disease and a well-characterized mouse model of human uveitis (17). We used this mouse model to investigate whether SBT-100 can be effective in suppressing the development or severity of this organ-specific CNS autoimmune disease. We induced EAU in C57BL/6J mice by immunization with IRBP_651-670_ in CFA emulsion as previously described (18). Starting from Day -1 of immunization to Day 12 postimmunization, mice were treated twice daily by i.p. injection with either 100 µL PBS or SBT-100 (10 mg/kg body weight in 100 µL PBS). In the EAU model, uveitis generally manifests between Day 13 and Day 22 postimmunization (p.i); therefore, we monitored progression and severity of the disease during this period by fundoscopy, histology, optical coherence tomography (OCT), and electroretinography (ERG). EAU clinical scores and assessment of disease severity were based on changes at the optic nerve disc or retinal vessels and detection of retinal and choroidal infiltrates in the eye. In this study, the initial signs of uveitis were first observed by Day 14 p.i., and full-blown inflammation was observed between then and Day 20 p.i. Fundus images of the PBS-treated retinae show severe inflammation characterized by blurred optic disc margins and enlarged juxtapupillary area, retinal vasculitis with moderate cuffing, and yellow-whitish retinal and choroidal infiltrates (**Figure 2A**). In contrast, fundus images of the SBT-100-treated mice retinae indicated a relatively mild EAU with very low clinical scores (**Figure 2A** and **Supplementary Figure 1**). Consistent with fundoscopy results, histology of the PBS-treated Day 21 retina revealed severe EAU with infiltration of large numbers of inflammatory cells into the vitreous, destruction of retinal cells, and development of retinal in-folding—a hallmark of severe uveitis (**Figure 2B**, red arrow). OCT is a noninvasive procedure for visualizing the microstructure of the retina and this procedure revealed accumulation of inflammatory cells in the vitreous and optic nerve head of the PBS-treated mice but not the retina of mice that received SBT-100 (**Figure 2C**). Electroretinogram (ERG) is a well-established clinical method for detecting alterations in visual function during intraocular inflammation and is based on changes in electrical potential in response to light stimulation of the retina (19, 20). ERG under light-adaptive stimuli reflect cone-driven functions while dark-adapted b-wave responses represent rod-driven activities. Thus, the significantly lower a- and b-wave amplitudes detected in the eyes of PBS-treated mice under light- or dark-adapted conditions, suggest significant visual impairment in PBS-treated mice while higher a- and b-wave amplitudes detected in SBT-treated mice are consistent with SBT-100 mediated preservation of vision during uveitis (**Figure 2D**).

### SBT-100 Suppressed Uveitis in Mice by Inhibiting Pathogenic Th1 and Th17 Cells

**Figure 3:**
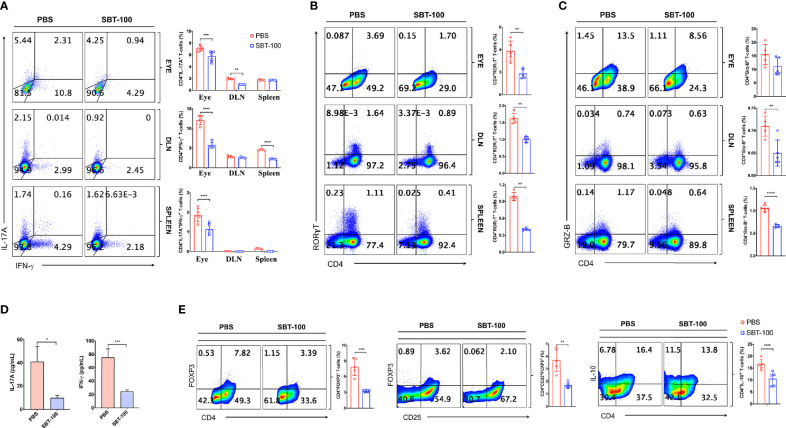
SBT-100 inhibits the differentiation and expansion of Th17 cells during EAU. C57BL/6J mice were immunized with IRBP_651-670_ in CFA and treated with PBS or SBT-100. **(A)** Percentage of Th1 or Th17 cells in the retina, DLN, or spleen, and Th1 or Th17 cells was determined by the intracellular cytokine staining. Quadrants indicate percentage of CD4^+^ T cells expressing IFN-γ or IL-17. **(B)** Percentage of CD4^+^ T cells expressing ROR-γT **(B)** or Granzyme B **(C)** in the retina, DLN, or spleen. **(D)** CD4^+^ T cells from the LN and spleen of EAU mice treated with PBS or SBT-100 were restimulated *in vitro* with IRBP_651-670_ peptide and IL-17A and IFN-γ secreted in Day 3 culture supernatant was detected by ELISA. **(E)** Cells from the spleen of the EAU mice treated with PBS or SBT-100 were analyzed by the intracellular cytokine staining. Quadrants indicate percentage of CD4^+^ T cells expressing Foxp3, CD25, and/or IL-10. Data represent at least three independent experiments and presented as mean ± SEM. (*p < 0.05; **p < 0.01; ***p < 0.001; ****p < 0.0001).

Uveitis in mice and humans is thought to be mediated primarily by Th17 and Th1 lymphocytes because levels of both T cell subsets are elevated in the eyes of mice with EAU and in uveitis and scleritis patients (7, 17, 21). Therefore, we investigated whether the amelioration of EAU observed in this study derived from SBT-100-induced decrease in levels of Th17 and/or Th1 cells. Our *in vitro* study showed that SBT-100 suppressed proliferation of primary T cells, suggesting that the number of CD4^+^ T cells would be reduced in mice treated with SBT-100. Therefore, in analysis of effect of SBT-100 on expansion of proinflammatory lymphocytes that mediate EAU, we used equal numbers of T cells from control (PBS-treated) and SBT-100-treated mice to examine frequencies of Th17 and Th1 cells in the eyes, draining lymph nodes, and spleen. We detected and quantified frequencies of CD4^+^ T cells secreting proinflammatory IL-17 and/or IFN-γ by the intracellular cytokine staining assay. Consistent with published reports, development of EAU in PBS-treated mice was accompanied by significant increase of IL-17-expressing (Th17) and IFN-γ-expressing (Th1) cells in the eyes, spleen, and DLN, while we observed much lower percentages of Th1 and Th17 in these tissues of mice treated with SBT-100 (**Figure 3A** and **Supplementary Figures 2A–C**). These results are in line with numerous studies implicating Th17 cells in pathogenesis of organ-specific autoimmune diseases (7, 8). Th17 differentiation and development requires ROR-γt transcription, and we show here that the decrease in Th17 cells correlated with detection of lower levels of this Th17 master transcription factor (**Figure 3B** and **Supplementary Figure 3A**). Th17 lymphocytes traffic to the CNS, mediate cytolytic effects *via* Granzyme B release, and blocking Granzyme B ameliorates late/chronic EAE (22, 23). Consistent with the curative effect of SBT-100, we observed a slight reduction of CD4 T cells producing Granzyme B in mice treated with SBT-100 compared to the untreated mice (**Figure 3C** and **Supplementary Figure 3B**). Measurement of IL-17 and IFN-γ in the supernatant of cultured CD4^+^ T cells from DLN was consistent with reduced levels of these cytokines in the SBT-100 treated mice (**Figure 3D**). Surprisingly, we found that SBT-100-mediated inhibition of EAU is not associated with increase of regulatory T cells (**Figure 3E** and **Supplementary Figure 4**).

### SBT-100 Suppresses Uveitis Induced by Adoptive Transfer of Uveitogenic Lymphocytes

**Figure 4:**
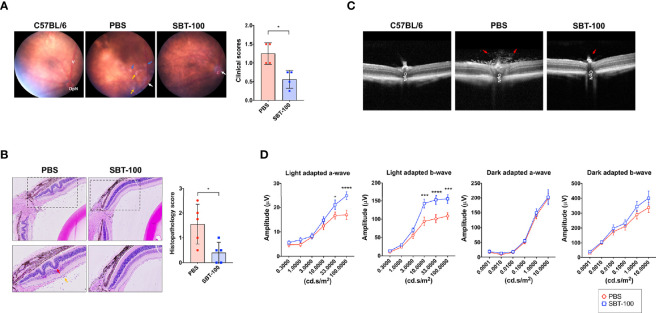
SBT-100 treated T cells are defective in transferring EAU to naive mice. T cells from lymph nodes and spleen of PBS-treated or SBT-treated mice with EAU were reactivated with IRBP_651-670_ peptide and adoptively transferred (i.p.) to unimmunized C57BL/6J mice. Twelve days after cell transfer, disease was assessed by fundoscopy **(A)**, histopathology **(B)**, OCT **(C)**, and ERG **(D)**. For the data shown, n = 5 mice. All data represent at least three independent experiments and presented as mean ± SEM. (*p < 0.05; ***p < 0.001; ****p < 0.0001).

We performed adoptive transfer experiments to directly investigate whether SBT-100 induced protection against EAU is a direct consequence of targeting the STAT3 pathway in T cells. We isolated cells from lymph nodes and spleen of control or SBT-100-treated C57BL/6J mice with EAU and reactivated the cells *ex vivo* with IRBP_651-670_ and adoptively transferred 3×10^7^ of the activated T cells to unimmunized C57BL/6J mice. We assessed progression of the disease and determined EAU scores 12 days after transfer of the cells by fundoscopy, histology, OCT, and ERG. We observed significant infiltration of inflammatory cells and vasculitis in mice that received cells from PBS-treated mice while mice that received cells from the SBT-100 treated mice were protected from severe EAU (**Figure 4A**). Retinal in-folding and cellular infiltration were also reduced in mice that received cells from SBT-100 treated mice (**Figure 4B**). OCT images also show significant accumulation of infiltrating cells around the optic nerve and distortion of retinal layers (**Figure 4C**). We also detected significantly higher light-adapted a- and b-waves, as well as substantial increase of dark-adapted b-waves in the eyes of mice that received cells from SBT-treated mice compared to mice that received cells from untreated mice (**Figure 4D**). These results indicate that the STAT3 pathway is a potential therapeutic target for ameliorating uveitis and preserved visual functions.

### SBT-100 Antagonized Expansion of Pathogenic Th1 and Th17 Cells That Mediate Uveitis

**Figure 5:**
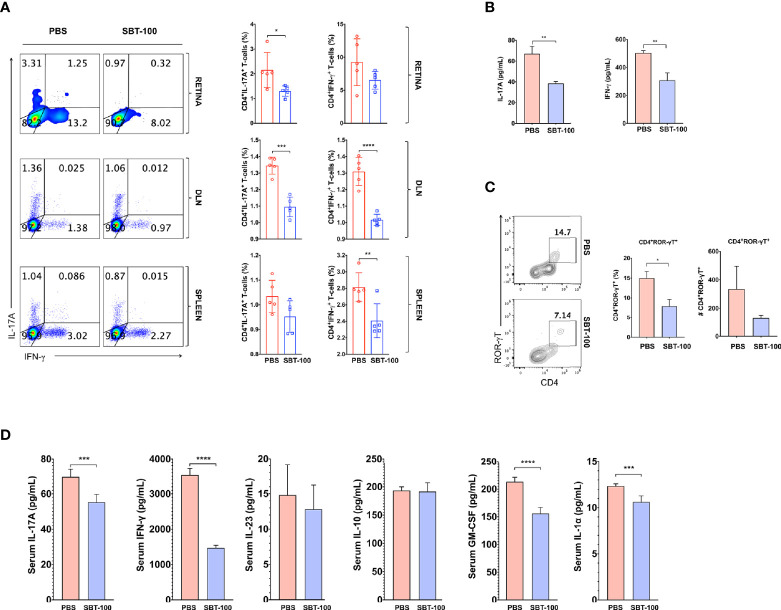
SBT-100 inhibits secretion of proinflammatory cytokines by pathogenic T cells. Following the adoptive transfer of activated IRBP-specific cells from PBS-treated or SBT-100 treated mice with EAU to naive C57/BL6J mice, we analyzed expression of Th1 and Th17 signature genes by intracellular cytokine staining assay or ELISA. Quadrants in **(A)** indicate percentage of CD4^+^ T cells expressing IFN-γ or IL-17 in the retina, DLN, or spleen and Th1 or Th17 cells. **(B)** Following the adoptive transfer of activated IRBP-specific T cells from PBS-treated or SBT-treated mice with EAU to naive C57/BL6J mice, CD4^+^ T cells from the lymph nodes and spleen of the recipient mice were restimulated *in vitro* with IRBP_651-670_ and IL-17A and IFN-γ secreted in Day 3 culture supernatant was detected by ELISA. **(C)** Quadrants indicate the percentage of CD4^+^ T cells expressing ROR-γT in the retina. **(D)** The levels of IL-17A, IFN-γ, GM-CSF, IL-1α, and IL-10 in the blood were detected by multiplex ELISA. Data represent at least two independent experiments and presented as mean ± SEM. (*p < 0.05; **p < 0.01; ***p < 0.001; ****p < 0.0001).

Like EAU induced by active immunization with IRBP_651-670_/CFA, EAU induced by the adoptive transfer method was also more severe in the untreated mice, indicating that targeting STAT3 pathway in T cells by SBT-100 conferred protection against EAU. Intracellular cytokine staining analysis shows that amelioration of EAU correlated with significantly reduced levels of pathogenic Th1 and Th17 cells in mice that received cells from mice treated with SBT-100 (**Figure 5A** and **Supplementary Figures 5A–C**). Thus, compared to untreated mice secretion of Th1 and Th17 signature cytokines, IFN-γ and IL-17, respectively, was significantly lower in SBT-100-treated mice with attenuated EAU (**Figure 5B**). Consistent with the well-established role of Th17 cell in the etiology of several autoimmune diseases, a percentage of T cells expressing the Th17 master transcription factor, (ROR-γt) is significantly reduced (**Figure 5C**). Analysis of the plasma of mice that received cells from untreated or SBT-100-treated mice revealed that the serum from the SBT-100 treated group has significant reduction of proinflammatory cytokines including IL-17, IFN-γ, IL-23, GM-CSF, and IL-1α, providing further suggestive evidence that SBT-100 treatment suppressed EAU (**Figure 5D**).

## Discussion

Uveitis is a diverse group of potentially sight-threatening intraocular inflammatory diseases that is characterized by repeated cycles of remission and recurrent intraocular inflammation, and visual handicap is of significant public health importance as it affects the patient’s quality of life. Increased recruitment of Th17 cells into the retina is implicated in pathophysiology of uveitis and current therapies include periocular or intravitreal corticosteroid. However, their prolonged use for treatment of chronic uveitis is associated with development of serious side effects such as glaucoma and is the impetus for developing alternative therapies. Targeting STAT3 pathway that is required for the differentiation and expansion of Th17 cells has been proposed as a potential therapy for mitigating uveitis because genetically modified mice that cannot induce Th17 cells are resistant to developing uveitis (7). However, a major impediment to targeting the STAT3 pathway is that it is an intracellular protein and not accessible to STAT3-specific antibodies and the unpredictable pharmacokinetic characteristics of small molecular weight STAT3 inhibitory peptides or mimetics.

**Figure 6:**
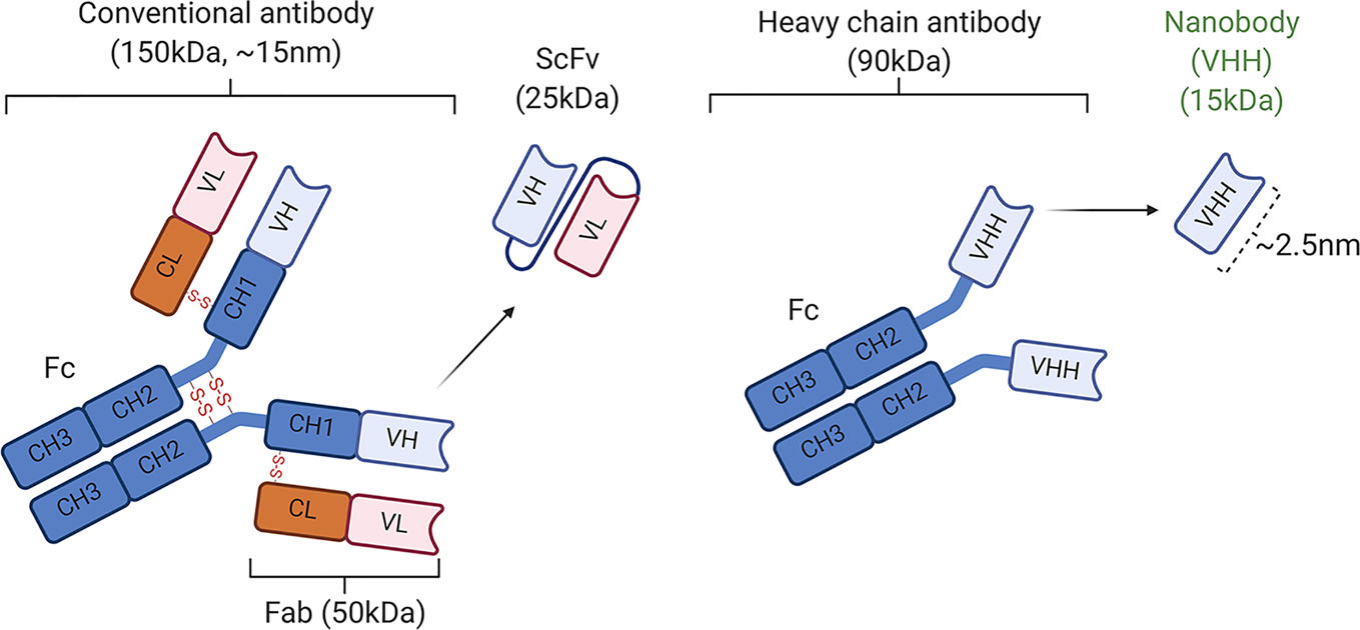
Schematic representation of conventional antibodies (IgG1) containing two light (L) chains (the VL and CL domains) and two heavy (H) chains and nanobodies (16). CH, constant domain of immunoglobulin H chain; Fab, antigen-binding fragment; Fc, crystallizable fragment; scFv, single-chain variable fragment.

In contrast to the conventional STAT3-specific antibodies composed of heterotetrameric immunoglobulins assembled from two identical heavy (H)-chain and two identical light (L)-chain polypeptides, the STAT3-specific nanobody we have used to target the STAT3 signaling pathway in this study is a unique camelid-like single-domain monomeric VHH antibody comprised of a unique one antigen-binding domain (**Figure 6**). In contrast to 90 kDa antibodies that do not penetrate cells, the STAT3 VHH is ~15 kDa (2.5 nm) in size, which allows it to penetrate cells and is not toxic to tissues. We induced uveitis in mice, treated them twice daily with SBT-100, and analysis of the eyes by fundoscopy, histology, optical coherence tomography, and electroretinography revealed that SBT-100 confers protection from severe uveitis and suppressed ocular inflammation by inhibiting Th1 and Th17 responses, and curtailed expansion and trafficking of inflammatory cells into retina during EAU. We further show that SBT-100 is not toxic to ocular cells, and it prevented decrement of retina function usually associated with severe ocular inflammation. Consistent with the well-established role of T cells in etiology of uveitis, we show that transfer of cells from EAU mice that were treated with SBT-100 induced very mild EAU compared to adoptive transfer of cells from untreated EAU mice that developed full-blown disease. Taken together, these results provide suggestive evidence that SBT-100 immunotherapy might be effective in ameliorating uveitis in humans.

CNS autoimmune diseases such as uveitis and multiple sclerosis result as a consequence of breakdown of immune privilege of the brain, spinal cord, or neuroretina, which are maintained by the BRB, BBB, and the NVU comprised of pericytes, perivascular macrophages, tightly bound endothelial cells, and glia limitans of the M̈ller/microglia (24, 25). These structures sequester CNS tissues from peripheral immune system and Th17 cells that produce Granzyme B are implicated in early events that initiate CNS autoimmune diseases by promoting the disruption of the BBB or BRB (11, 26–28). However, sustained activation of microglial cells and recruitment of other inflammatory cells amplify the inflammatory response and are responsible for pathology characteristic of chronic uveitis or multiple sclerosis. Nonetheless, interventional studies using biologics such as cytokines or immune-suppressive compounds to suppress uveitis in mice invariably show strong correlation of disease amelioration with suppression of pathogenic Th17 cells (17, 21). Subsequent studies revealed the requirement of STAT3 for Th17 differentiation and development while others showed that targeted deletion of STAT3 prevented the development of EAE or EAU. These studies led to the now-established notion that targeting Th17 cells is a viable therapeutic approach for suppressing and mitigating autoimmune and autoinflammatory diseases.

In this study, SBT-100 immunotherapy conferred protection against EAU by antagonizing the expansion of the pathogenic Th1 and Th17 cells but also Treg cells. While Th17 cells play an important role in initiating the disease, our data suggest that therapeutics designed to inhibit Th17 would only be partially effective. Our data is consistent with previous studies showing that STAT3 has a dual role in T cells: it plays the important role of maintaining unactivated T cells at the G_0_ phase as resting cells by inhibiting IL-2 production through up-regulation of the lymphocyte quiescence Class O Forkhead transcription factors (5, 6). It is of note that, while STAT3 maintains a T cell as resting cells, after engaging cognate antigen and entry into the G_1_ cell cycle phase, STAT3 promotes cell proliferation as is the case in all mammalian cells. Thus, the STAT3-specific nanobody mediates suppression of Th17 and Th1 lymphocytes that initiate or perpetuate neuroinflammation and the immunosuppressive Tregs. However, we were not surprised that Treg levels were also affected by SBT-100 as STAT3 is required for proliferation of all mammalian cells. Although the suppression of Tregs by SBT-100 may raise concern of inducing generalized suppression, it is of note that the standard of care for uveitis is topical or systemic corticosteroids that can induce generalized immune suppression and other deleterious effects. As is the case with steroids, antibiotics, or biologics including anti-INF-γ, anti-IL-2 (daclizumab), TNF-α (etanercept, infliximab, adalimumab), IL-12/IL-23 antagonist (ustekinumab) that have also been used in uveitis, these drugs are for short-term use. Moreover, the retina is an immune privileged tissue endowed with multiple and redundant immunosuppressive mechanisms that actively suppress inflammation and reduction of IL-10 level is of less concern.

In summary, uveitis is a group of syndromic diseases which includes sympathetic ophthalmia, birdshot retinochoroidopathy, Behcet’s disease, Vogt-Koyanagi–Harada disease, and ocular sarcoidosis (29, 30). It accounts for more than 10% of severe visual handicaps in the United States and a major impediment to treatment of the disease with antibodies is their size, which restricts entry into the CNS because of the BRB and the neuroretinal vascular unit. We have shown for the first time that a single domain antibody, STAT3-specific VHH, can cross the BRB, treat an organ-specific autoimmune ophthalmic disease, and significantly inhibit both Th17 and Th1 cells *in vivo*. Moreover, the STAT3 specific nanobody is a nontoxic antibody that readily enters CNS tissues such as the brain, spinal cord, and the neuroretina. Thus, the STAT3-specific VHH provides a template for designing antibodies that can be used to suppress undesirable activities of neuronal proteins of the CNS.

## Data Availability Statement

The raw data supporting the conclusions of this article will be made available by the authors, without undue reservation.

## Ethics Statement

The animal study was reviewed and approved by National Institute of Health guidelines (NIH Animal Care and Use Committee guidelines).

## Author Contributions

EM performed most studies, prepared the figures, and edited the manuscript. MY performed immunization of mice. SA assisted with EAU studies. MK assisted with EAU experiments. YJ performed EAU disease scoring, fundoscopy and ERG. C-RY assisted with EAU studies. AP characterized SBT-100 nanobody. SS designed, produced, and characterized the SBT-100 nanobody. CE conceived, designed, and supervised the project, and wrote the manuscript. All authors contributed to the article and approved the submitted version.

## Conflict of Interest

AP and SS manufactured the SBT-100 nanobody and have proprietary rights to the use of SBT-100. SS was employed by Singh Biotechnology.

The remaining authors declare that the research was conducted in the absence of any commercial or financial relationships that could be construed as a potential conflict of interest.

## Publisher’s Note

All claims expressed in this article are solely those of the authors and do not necessarily represent those of their affiliated organizations, or those of the publisher, the editors and the reviewers. Any product that may be evaluated in this article, or claim that may be made by its manufacturer, is not guaranteed or endorsed by the publisher.
