# Supplementary material for: STAT3-Specific Single Domain Nanobody Inhibits Expansion of Pathogenic Th17 Responses and Suppresses Uveitis in Mice
Source: Front Immunol. 2021 Sep 15;12:724609. doi: 10.3389/fimmu.2021.724609 (PMC8479182; doi:10.3389/fimmu.2021.724609)
Supplement: Supplementary file 1 [file DataSheet_1.pdf]

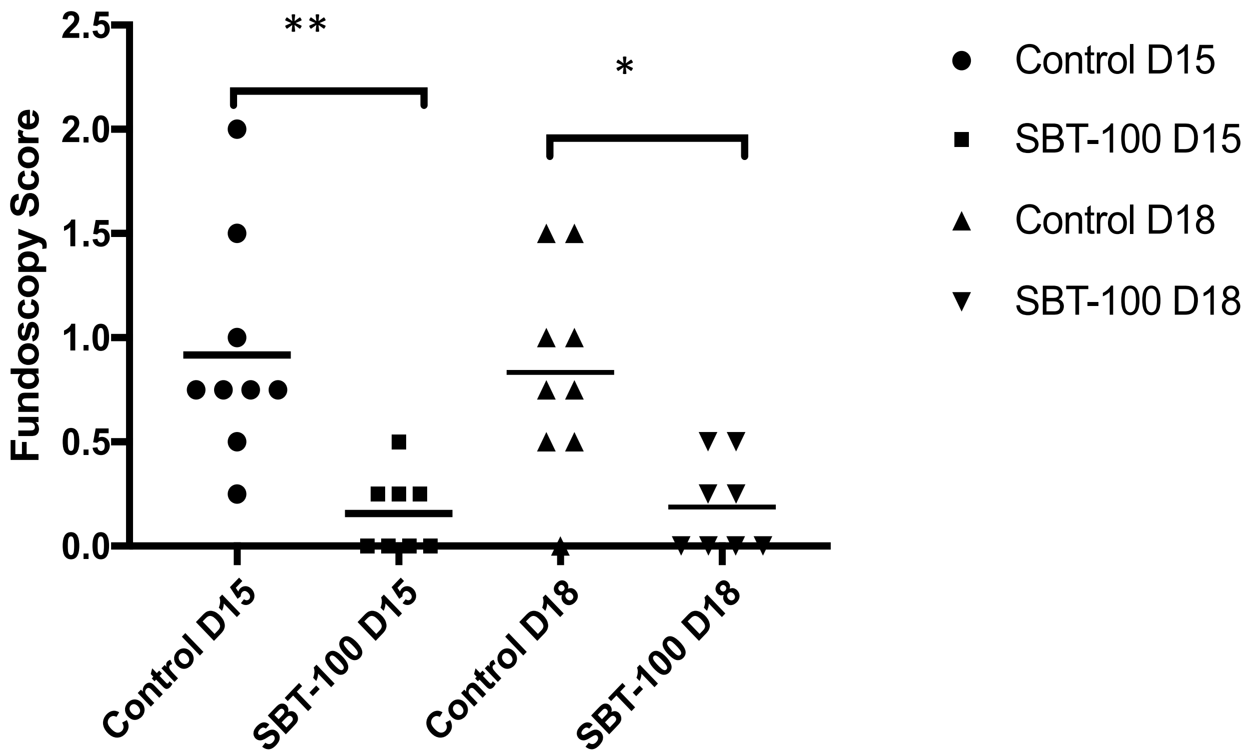

Supplementary Figure 1. C57BL/6J mice were immunized with IRBP in CFA and treated with PBS or SBT-100 and development of EAU was assessed by fundoscopy. Clinical scores and assessment of disease severity were based on changes at the optic nerve disc or retinal vessels and retinal and choroidal infiltrates, following scores guidelines previously reported. Data represent an additional independent experiment and presented as mean ± SEM. (*$p < 0.05$; **$p < 0.01$).

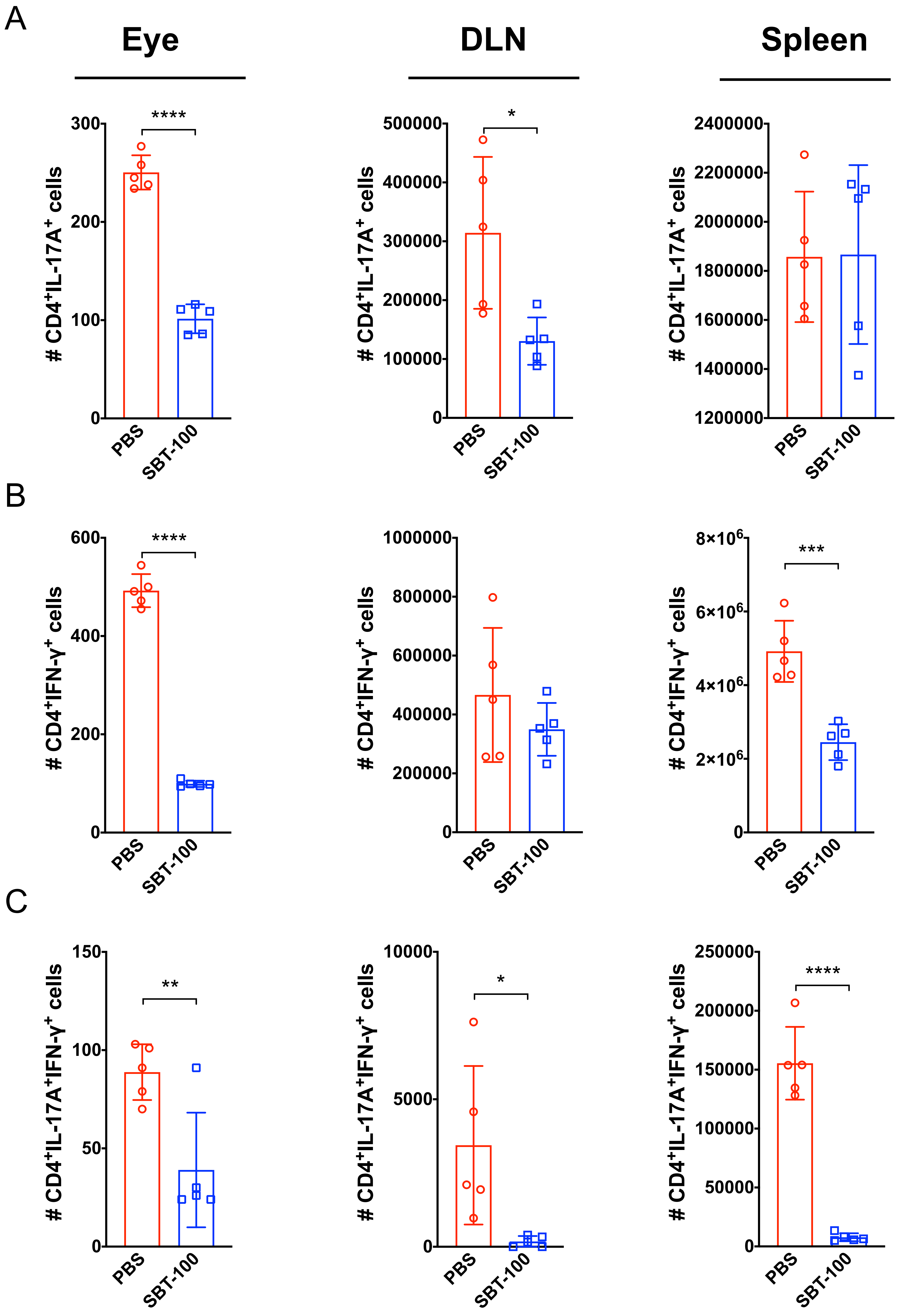

Supplementary Figure 2. EAU was induced in C57BL/6J mice by immunizing with IRBP in CFA and treated with PBS or SBT-100. Absolute numbers of $CD4^{+}IL\text{-}17^{+}$ (A), $CD4^{+}IFN\text{-}\gamma+$ (B) and $CD4^{+}IFN\text{-}\gamma^{+}IL\text{-}17A^{+}$ double positive T cells in the eye, draining lymph node or spleen. p-values (*p < 0.05, **p < 0.01, ***p < 0.001, ****p < 0.0001).

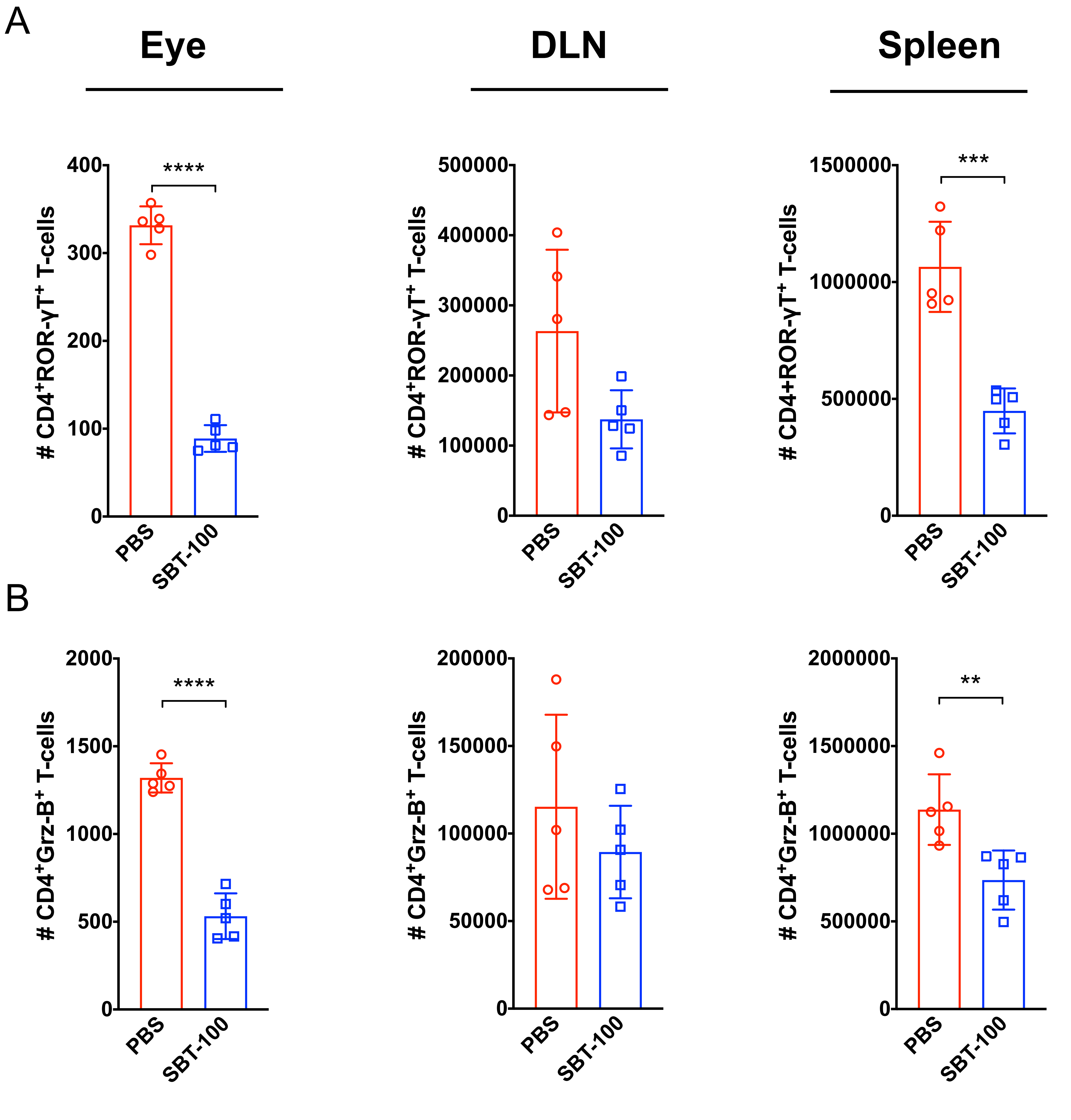

Supplementary Figure 3. EAU was induced in C57BL/6J mice by immunizing with IRBP in CFA and treated with PBS or SBT-100. Absolute numbers of $CD4^{+}ROR\text{-}\gamma T^{+}$ (A), $CD4^{+}Grz\text{-}B^{+}$ (B) T cells in the eye, draining lymph node or spleen. Asterisks in figures denote p-values (**$p < 0.01$, ***$p < 0.001$, ****$p < 0.0001$).

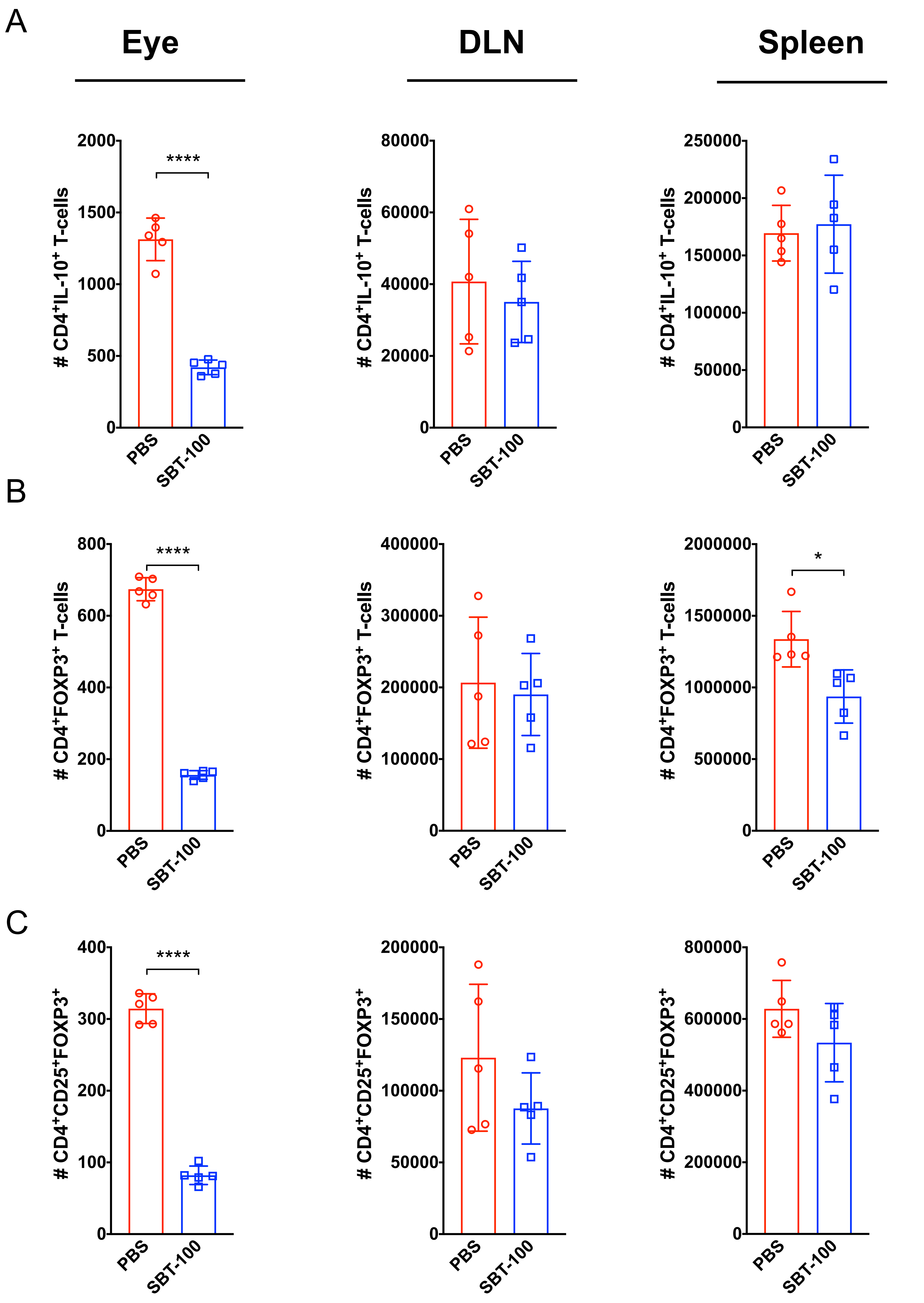

Supplementary Figure 4. EAU was induced in C57BL/6J mice by immunizing with IRBP in CFA and treated with PBS or SBT-100. Absolute number of $CD4^{+}IL\text{-}10^{+}$ (A), $CD4^{+}FOXP3^{+}$ (B) and $CD4^{+}FOXP3^{+}CD25^{+}$ (C) T cells in the eye, draining lymph node or spleen. Asterisks denote p-values (*$p < 0.05$, ****$p < 0.0001$).

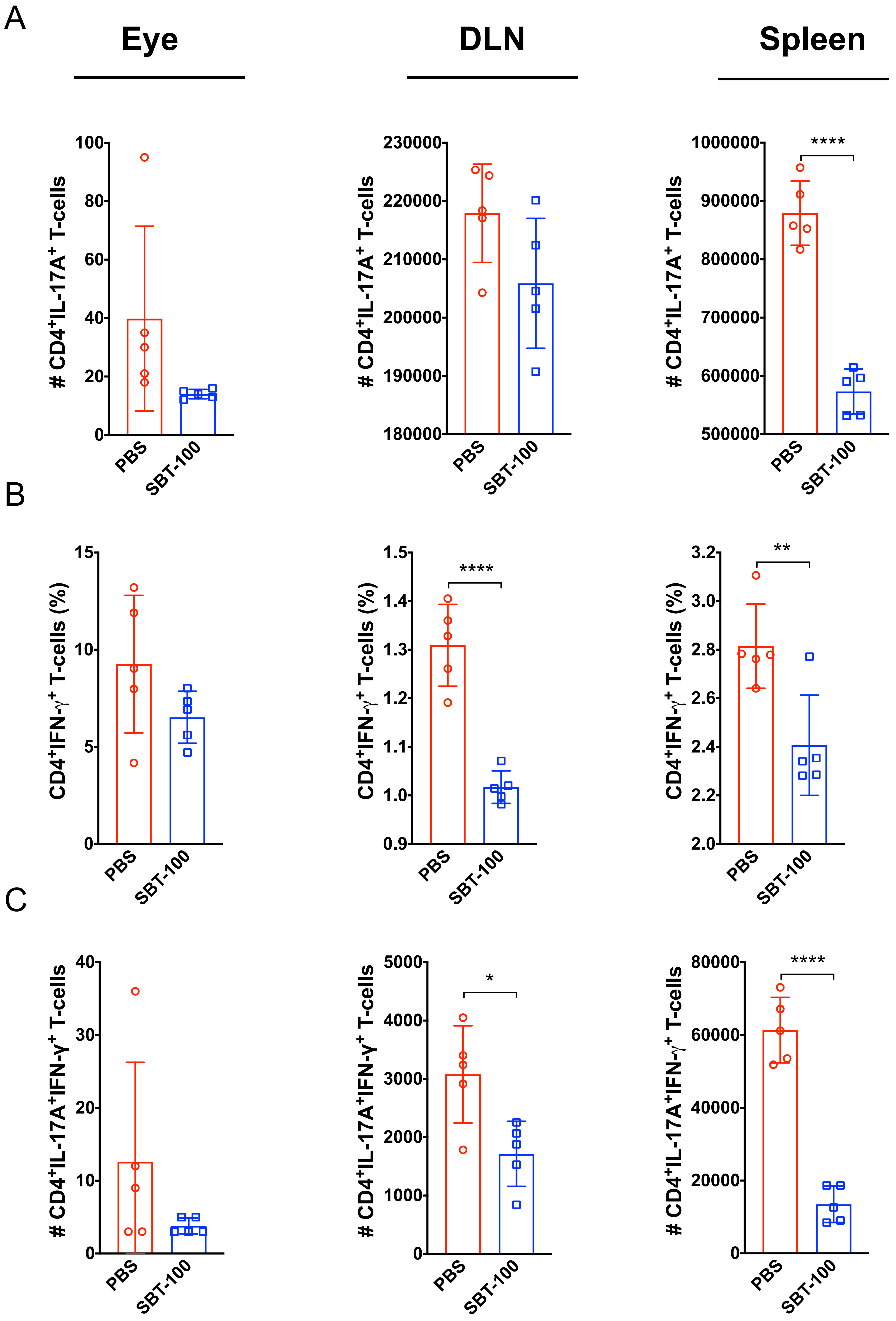

Supplementary Figure 5. Adoptive transfer of IRBP reactivated DLN cells from PBS-treated or SBT-100-treated mice with EAU to naïve C57BL/6J mice. Absolute number of $CD4^+$ T cells expressing IL-17A (A), IFN-γ (B) or $CD4^+IFN\text{-}\gamma^+IL\text{-}17A^+$ (C) double positive T cells in the eye, draining lymph node or spleen. Asterisks denote p-values (*$p < 0.05$, ****$p < 0.01$, ****$p < 0.0001$).
